# European common frog (*Rana temporaria*) recolonized Switzerland from multiple glacial refugia in northern Italy via trans‐ and circum‐Alpine routes

**DOI:** 10.1002/ece3.8268

**Published:** 2021-11-02

**Authors:** Alexandra Jansen van Rensburg, Mathieu Robin, Barret Phillips, Josh Van Buskirk

**Affiliations:** ^1^ Department of Evolutionary Biology and Environmental Studies University of Zurich Zurich Switzerland; ^2^ Centre for Biodiversity and Environmental Research University College London London UK; ^3^ Department of Ecology and Evolution University of Lausanne Lausanne Switzerland

**Keywords:** European Alps, glacial refugia, phylogeography, *Rana temporaria*

## Abstract

The high mountain ranges of Western Europe had a profound effect on the biotic recolonization of Europe from glacial refugia. The Alps present a particularly interesting case because they form an absolute barrier to dispersal for most taxa, obstructing recolonization from multiple refugia in northern Italy. Here, we investigate the effect of the European Alps on the phylogeographic history of the European common frog *Rana temporaria*. Based on partial *cytochrome b* and *COXI* sequences from Switzerland, we find two mitochondrial lineages roughly north and south of the Alpine ridge, with contact zones between them in eastern and western Switzerland. The northern haplogroup falls within the previously identified Western European haplogroup, while the southern haplogroup is unique to Switzerland. We find that the lineages diverged ~110 kya, at approximately the onset of the last glacial glaciation; this indicates that they are from different glacial refugia. Phylogenetic analyses suggest that the northern and southern haplogroups colonized Switzerland via trans‐ and circum‐Alpine routes from at least two separate refugia in northern Italy. Our results illustrate how a complex recolonization history of the central European Alps can arise from the semi‐permeable barrier created by high mountains.

## Graphical abstract

The European Alps presented a barrier to the post‐glacial recolonization of Europe for many taxa. However, a few cold‐tolerant species were able to colonize Switzerland across the Alps via multiple colonization routes from refugia in Northern Italy. We show that the complex colonization history of *Rana temporaria* across the Alps has resulted in unusually high genetic diversity within Switzerland.

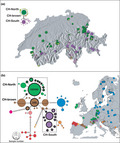

## 1 INTRODUCTION

High mountain ranges such as the European Alps and Pyrenees were the last to deglaciate in mainland Europe during glacial minima over the last ~700 ky (Darnault et al., 2012; Ehlers et al., 2018). For taxa adapted to warm climates, the persistence and extent of Alpine ice sheets and the east–west orientation of the mountain ranges hindered recolonization from more southerly refugia in Italy and the Iberian Peninsula into Europe (Taberlet et al., 1998). Cold‐tolerant organisms exhibit a more complex history. Many expanded their range during glacial periods, some persisted in nunataks (e.g., ice‐free rocky protrusions) within the Alps, and trans‐Alpine recolonization has occurred in several species (e.g., Oak species, the common shrew, and barred grass snake, and the European stag beetle; Mátyás & Sperisen, 2001; Lugon‐Moulin & Hausser, 2002; Parisod, 2008; Kindler & Fritz, 2018; Cox et al., 2019). However, the effect of the Alps on the biogeography of cold‐tolerant species remains poorly understood, particularly for vertebrates (but see Braaker & Heckel, 2009; Yannic et al., 2008).

Here, we describe the phylogeographic history of a cold‐tolerant amphibian, the European common frog (*Rana temporaria*), by densely sampling populations across the Alps in Switzerland. This species is the most widespread anuran in Europe, occurring from northern Italy and Spain to the sub‐arctic tundra in Fennoscandia in the north and the Ural mountains in the east. *R*. *temporaria* is ubiquitous in the European Alps, breeding up to an elevation of 2600 m (Sillero et al., 2014). Two deeply diverged (~0.7 Mya) mitochondrial lineages occur in Western and eastern/northern Europe, respectively (Palo et al., 2004). A contact zone has been identified that extends from northern Germany (Schmeller et al., 2008) to southern France, the northern lowlands of Switzerland, and northwestern Italy (Teacher et al., 2009). The precise location and structure of the contact zone in the Alps has not been resolved (Rodrigues et al., 2017; Stefani et al., 2012), although this will be particularly interesting because it reflects the phylogeographic history and the distribution of genetic diversity in this region. The western haplogroup has higher genetic diversity than the eastern haplogroup and sex chromosome differentiation parallels the distribution of mitochondrial lineages (Phillips et al., 2020), suggesting that *R*. *temporaria* recolonized Western Europe from multiple glacial refugia. Both Iberia (Teacher et al., 2009) and Italy (Stefani et al., 2012) have been suggested as principal refugia for the western haplogroup. However, sampling has not been available to reconstruct the main routes of post‐glacial recolonization of the western haplogroups, and it remains unclear how the Alps influenced recolonization into Western Europe.

Our main aims are to determine the colonization history and extant population structure of *Rana temporaria* in Switzerland. Specifically, we ask whether (1) a contact zone exists between Eastern and Western haplotypes in Switzerland, (2) Switzerland was colonized from one or multiple glacial refugia, and (3) the Alps were a barrier to colonization.

The geography of the Alps between northern Italy and Switzerland is characterized by two main high mountain ridges running roughly east to west. We refer to the first as the southern Alpine ridge. This ridge includes the highest peaks and roughly constitutes the border between Italy and Switzerland. The second ridge is called the northern Alpine ridge, and roughly bisects Switzerland into a northern and southern part.

## 2 MATERIALS AND METHODS

### 2.1 Sampling

**FIGURE 1:**
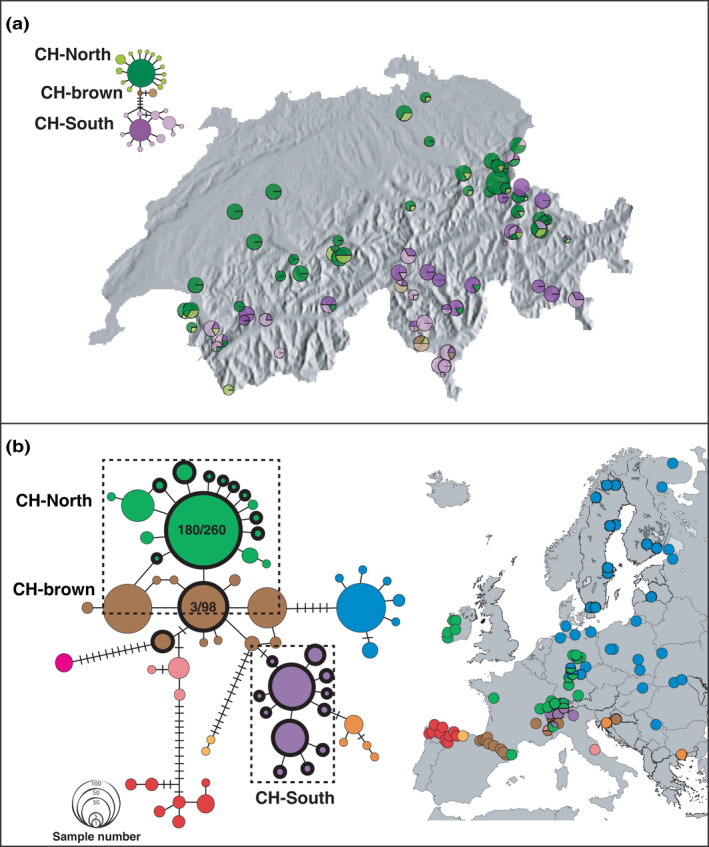
Geographic distribution of *cytb* haplotypes in Europe and Switzerland. (a) *Cytb* (448 bp) haplotypes from Switzerland. The inset depicts the *cytb* haplotype network calculated for all Swiss samples (368 individuals from 72 populations sequenced at 448 bp). The most common haplotype within each haplogroup is shaded in a darker color compared to the derived haplotypes. (b) *Cytb* (331 bp) of concatenated dataset of data from this study and Vences et al. (2013). Geographic distribution of the haplotypes within the eastern (blue) and western (other colors) clades is shown on a map of Europe. Swiss haplotypes (shown with bold outlines) fall within the green (CH‐North) and brown (CH‐brown) haplogroups, and a Swiss‐specific purple haplogroup (CH‐South). Numbers in the green and brown haplotypes show the proportion of samples from this study assigned to that haplotype

To investigate phylogeographic patterns and the geographic distribution of genetic variation, we sampled 82 populations across a ~2300 m gradient of elevation in Switzerland during the 2013 breeding season (Figure 1a, Table S1). Approximately, 10 eggs from each of 20–30 freshly laid clutches were collected from each site. The eggs were transported to the University of Zürich, where they were hatched in separate water‐filled containers in a water bath. Tadpoles were euthanized and stored in ethanol when they reached stage 36 (Gosner, 1960). One individual per clutch was used for all work presented here. Ethical permits were issued by the Veterinary Office of the Canton Zürich (authorization 61/2013_4946). In addition, we obtained tissue samples of 40 clutches from 10 populations in northern Italy, Serbia, and Germany to complement the Europe‐wide *cytochrome b* sequences that are publicly available (Table S2).

### 2.2 Molecular lab work

Total DNA was extracted from tadpole tails by overnight digestion in 10% proteinase K solution at 56°C and extracted using the Qiagen Biosprint 96 DNA blood kit (Qiagen, CA, USA). DNA was eluted in 100–200 μl buffer AE (QIAgen).

To establish phylogeographic patterns at a European scale, we sequenced the same section of the *cytb* gene that was studied in earlier work on *R*. *temporaria* (Teacher et al., 2009; Vences et al., 2013). A 448 bp fragment of *cytb* was amplified by PCR using *Rana*‐*cytb*‐*F2* (5′ TTAGTAATAGCCACAGCTTTTGTAGGC) and *Rana*‐*Cytb*‐*R2* (5′ AGGGAACGAAGTTTGGAGGTGTGG) primers (Vences et al., 2013) with an annealing temperature of 53°C.

Teacher et al. (2009) and Vences et al. (2013) did not sample the Italian Alps at a fine scale. Therefore, we generated sequence data to compare with Italian samples from Stefani et al. (2012). We PCR amplified a 628bp fragment of COXI. Primers L4437 (5′ AAGCTTTCGGGCCCATACC) and H6564 (5′ GGGTCTCCTCCTCCAGCTGGGTC) with an annealing temperature of 49°C for a subset of 44 populations were collected from Switzerland.

Amplified COXI and *cytb* fragments were subjected to Sanger sequencing after we purified them using a standard ExoSap protocol: 0.25 μl Exonuclease I (20 U/μl; New England Biosystems), 0.5 μl rAPID Alkaline Phosphatase (SAP) (1 U/μl), 7.25 μl nuclease‐free water, and 8 μl PCR product. Cycling conditions were as follows: 37°C for 45 min, 80°C for 15 min, and held at 10°C. From the cleaned product, 2 μl per sample was used in 10 μl sequencing reactions (BigDye Sequencing Kit, Applied Biosystems). Samples were sequenced in one direction for each gene using an automated 3130xl DNA Analyzer (Applied Biosystems) and the sequences were aligned using CLC Main Workbench 5.0.2 (CLC Bio) and BioEdit (Hall, 1999). Sequences were verified as *cytb* and COXI using the BLASTN 2.2.24 algorithm (Altschup et al., 1990) implemented on the National Center for Biotechnology Information (NCBI) online platform.

Palo et al. (2004) identified a restriction enzyme cut site at position 277 in the *cytb* sequence that distinguishes between the eastern and the western mitochondrial clades. We used this assay to assign our samples to clades. Restriction digests were performed using 8 μl of the PCR product, 0.1 μl 10,000 U/ml *StyI* restriction enzyme (New England Biolabs), 0.9 μl water, and 1 μl 10× Buffer (Qiagen). PCR products were digested for 30 min at 37°C and then resolved on a 1% agarose gel and assessed by eye under UV light.

### 2.3 Analysis

#### 2.3.1 Geographic distribution of mitochondrial variation

To visualize the geographic distribution of genetic variation, haplotype networks were constructed with *cytb* and COXI sequences using statistical parsimony (Templeton et al., 1992) as implemented in TCS version 1.21 (Clement et al., 2000). Within Switzerland, we used the 368 individuals sequenced at 448bp *cytb* and 151 individuals sequenced at 1076bp (628bp COXI and 448bp *cytb*). At a broader geographic scale, we visualized genetic variation by combining our data with those from elsewhere in Europe obtained from NCBI (accession numbers for *cytb*: KC799522.1–KC800122.2; Vences et al., 2013). This dataset comprised 969 individuals sequenced at 331 bp *cytb*. These data did not include Italian samples described in Stefani et al. (2012) because a different *cytb* fragment was amplified in their study. Phylogenetic relationships between Swiss and Italian samples were explored separately using COXI (see below).

To estimate the geographic distribution of genetic diversity, we calculated haplotype diversity (*H*) and nucleotide diversity (*π*) using DnaSP 5.0 (Librado & Rozas, 2009). This was done for all Swiss populations combined and separately for the two lineages occurring north and south of the main Alpine ridge.

#### 2.3.2 Phylogenetic analyses

Evolutionary relationships among geographic groups in the haplotype network were determined with phylogenetic analyses on three datasets: *cytb1*, COXI, and the two combined. We included data from this study and all available data from Vences et al. (2013). Outgroup species were chosen based on the *Rana* phylogeny in Veith et al. (2003) and data available from NCBI. For *cytb*, the outgroups were *R*. *pyrenaica* (KC799521.1), *R*. *arvalis* (KC800123), *R*. *iberica* (KC799485), and *R*. *italica* (KC799493). For COXI, the outgroups were *R*. *pyrenaica* (KC977251.1) and *R*. *arvalis* (JN971596.1).

Our first step was to identify the evolutionary mutation model that best fit each dataset by comparing 88 alternative models in jModeltest2 (Darriba et al., 2012) and using the Akaike Information Criterion (Akaike, 1973) to select the model best supported by the data. Next, we conducted phylogenetic analyses using maximum likelihood and Bayesian methods. Maximum likelihood analyses were implemented in RAxML v.8.2.2 (Stamatakis, 2006). For each dataset, 1000 rapid bootstrap replicates were conducted followed by a maximum likelihood search for the most likely phylogenetic tree. Bayesian phylogenetic analyses were conducted using MrBayes v.3.2.1 (Huelsenbeck & Ronquist, 2001). Analyses were run for 1 × 10^6^ generations and sampled every 1000 generations. Four Metropolis‐coupled Markov chain Monte Carlo chains were used for two replicate runs of each dataset, with chain heating kept at default temperatures. We confirmed that effective sample sizes for each parameter were adequate (>200), chains mixed appropriately, and the average standard deviation of split frequencies between independent runs was <0.05. A burnin of 25% was used to obtain the consensus phylogram and posterior probabilities for each bipartition. The two approaches yielded the same outcome, thus we present only the Bayesian results here.

We estimated the coalescence date for all *R*. *temporaria* haplotypes and the Swiss subset of haplotypes using a Bayesian Markov chain Monte Carlo analysis with *R*. *arvalis* and *R*. *pyrenaica* as outgroups (BEAST v. 1.8.2; Drummond et al., 2012). COXI and *cytb* were analyzed as a single dataset, totaling 1076 bp, because jModeltest inferred a similar nucleotide substitution model for both gene fragments. We used the GTR +I substitution model, with estimated base frequencies, and six nucleotide substitution rate categories. Priors were set to their defaults except for the date of divergence of the three *Rana* species, which was specified as a lognormal distribution with a mean of 3.18 Mya and standard deviation of 0.43 Mya (Veith et al., 2003). Tests were run with all combinations of demographic and molecular clock preset priors available in BEAST, and the most likely model was selected based on a marginal likelihood test. The final analysis was run using a relaxed molecular clock, and a demographic model of constant population size.

#### 2.3.3 Origin of haplotypes in Switzerland

To investigate potential colonization routes between the Italian Alps and Switzerland, we estimated the phylogenetic relationship between 569 bp COXI data from this study and the corresponding data available on NCBI. This analysis was based on COXI only because the publicly available *cytb* fragment sequenced for samples from northern Italy (Stefani et al., 2012) does not overlap with that of samples from Europe (Vences et al., 2013). We included *R*. *temporaria* haplotypes from the Italian Alps (Stefani et al., 2012; FN813783–FN813812) and Europe (Vences et al., 2013; KC977228.1–KC977251.1), and selected the sister taxa *R*. *pyrenaica* (KC977251.1) and *R*. *arvalis* (JN971596.1) as outgroups. Phylogenetic analyses were conducted as described above.

#### 2.3.4 Demographic history

To test for recent changes in population size, we used Tajima's *D*, Fu and Li's *D*, Fu's *F*, and the DNA sequence mismatch distribution (Rogers & Harpending, 1992). A mismatch distribution tests for signatures of population expansion based on the number of nucleotide differences (mismatches) between DNA sequences. The distribution of mismatches is expected to be multimodal when population size remains stable, reflecting the stochastic nature of gene trees. When populations expand, a unimodal distribution of mismatches is expected.

## 3 RESULTS

The dataset comprised 368 families from 72 populations sequenced at 448 bp of the *cytb* gene, and 151 families from 44 populations sequenced at 628bp of COXI. Based on the absence of the diagnostic *cytb* restriction site, all Swiss samples were determined to be from the western clade.

### 3.1 Geographic distribution of mitochondrial variation

The haplotype network revealed eastern (blue) and western (other colors) clades with much higher genetic diversity found in the western clade (Figure 1b). The Swiss samples assigned to three western haplogroups: (1) The CH‐North haplogroup corresponded to the widespread Western European haplogroup, which is thought to have colonized Western Europe from the Pyrenees (green haplotypes in Figure 1; Vences et al., 2013). (2) CH‐brown is closely related to the widespread Western European haplogroup, and occurs almost exclusively in the Pyrenees (brown haplotypes in Figure 1; Vences et al., 2013). (3) The southern haplogroup (CH‐South) was unique to Switzerland, and fell between the haplogroups sampled in the Pyrenees and those sampled in Croatia and Greece in the haplotype network. CH‐North and CH‐South co‐occurred in some populations, across all elevations in eastern Switzerland and at low elevation in western Switzerland, while CH‐brown co‐occurred with CH‐South haplotypes in southern Switzerland (Figure 1a). This division was confirmed when Swiss samples were analyzed using cytb alone and in combination with COXI (Figure S1).

Genetic diversity within Switzerland was higher in populations found south of the Alpine ridge. Haplotype and nucleotide diversity based on 448 bp *cytb* gene fragment were roughly double in CH‐South when compared to CH‐North populations, and three times higher based on 628 bp COXI sequence (Table 1; diversity measures for individual populations are reported in Tables S1 and S3).

**TABLE 1:** Diversity measures and neutrality tests for the *cytb*, COXI, and concatenated datasets generated in this study

Pop group	*N*	Length (bp)	nr Hap	Hd (SD)	*π* (SD)	Tajima's *D*	Fu & Li's *D*	Fu & Li's *F*
*cytb*								
All	368	448	29	0.712 (0.020)	0.00857 (0.00019)	−0.377	−2.896*	−2.202
CH‐North	217	448	17	0.347 (0.042)	0.00126 (0.00021)	−2.207**	−3.945**	−3.918**
CH‐South	151	448	12	0.633 (0.034)	0.00249 (0.00016)	−1.068	−1.429	−1.550
*COXI*								
All	151	628	15	0.614 (0.033)	0.00517 (0.00023)	−0.1242	−1.797	−1.381
CH‐North	92	628	7	0.167 (0.053)	0.00031 (0.00011)	−2.039*	−3.117*	−3.264**
CH‐South	59	628	8	0.485 (0.078)	0.00103 (0.00021)	−1.468	−1.179	−1.497
*Concatenated cytb +COXI*								
All	141	1076	31	0.792 (0.029)	0.00658 (0.00026)	−0.067	−2.848	−2.034
CH‐North	86	1076	17	0.557 (0.065)	0.00080 (0.00014)	−2.209***	−3.415**	−3.545**
CH‐South	55	1076	14	0.706 (0.064)	0.00157 (0.00018)	−1.194	−2.147	−2.158

Note

CH‐North includes all samples with haplotypes from the Northern haplogroup, and CH‐South from the Swiss‐specific haplogroup. Three tests for selection are included. * = *p* < .05; ** = *p* < .01; *** = *p* < .001.

*p < .05, **p < .02, ***p < 0.

Abbreviation: *H*_d_, haplotype diversity (standard deviation);length, sequence length in base pairs; *N*, Number of samples; *N*_d_, nucleotide diversity (standard deviation); nr Hap, the number of haplotypes detected.

### 3.2 Phylogenetic analyses

**FIGURE 2:**
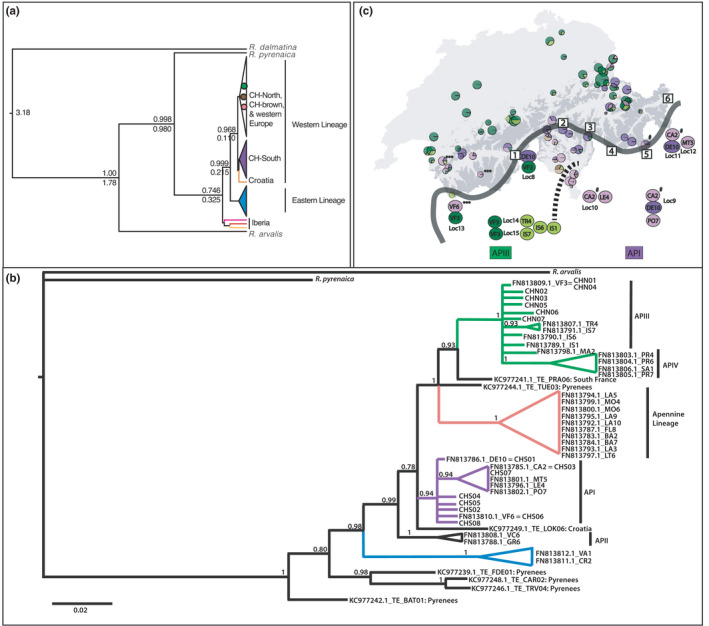
Bayesian phylogenetic tree of mitochondrial haplotypes from this and other studies, and their geographic distribution. Colored regions match the haplogroups identified in Figure 1b. Haplotypes from this study are indicated with CHN (CH‐North) and CHS (CH‐South). (a) Concatenated *cytb* and *COXI* genes. Posterior probability is shown above the line, and the inferred coalescent time (Mya) is shown below the line. (b) COXI haplotypes (569 bp) sampled across the *R. temporaria* range. Italian lineages described by Stefani et al. (2012) (Alpine lineage I–IV and Apennine lineage; NCBI code FNxx) are shown with vertical black bars. Four haplotypes from this study match Stefani et al. (2012) haplotypes and are indicated with an equal (=) sign. CH‐brown haplotypes are not present in this figure as these populations were not sequenced at COXI. Haplotypes from Vences et al. (2013) are shown with their NCBI codes (KCxx) and their sample locations. Posterior probability of >0.5 is shown above each node. The scale bar shows the number of expected substitutions per site. (c) Possible trans‐alpine colonization routes from the Italian Alps into Switzerland. The map shows elevation >2000 m in dark gray; thus, the light gray highlights the potential regions available for colonizing populations. The thick solid gray line roughly denotes the central Alpine ridge. We show the geographic distribution of the COXI haplotypes identified by Stefani et al. (2012) in northern Italy bordering Switzerland. The dashed line indicates the Ticino valley that roughly separates API (light and dark purple) from APIII (light and dark green). For illustration purposes, the haplotypes shown in Switzerland are from *cytb* sequences (i.e., Figure 2a) since mitochondrial haplotypes are linked, and we sequenced this gene more extensively than COXI (see Figures S2 and S3 for COXI). The Italian populations are defined as in Stefani et al. (2012); Loc8: Pramollo (46.36 N 11.86 E), Loc9: M.Pora (45.88 N 10.1 E), Loc10: Lemna (45.85 N 9.18 E), Loc11: S.Caterina (46.41 N 10.49 E), Loc12: V.Martello (46.49 N 10.69 E), Loc13: V.Ferret (45.85 N 7.02 E), Loc14: Traversella (45.53 N 7.72 E), Loc15: Issiglio (45.46 N 7.32 E). Four haplotypes in Stefani et al. (2012) matched sequences from this study and their geographic locations are shown as follows: VF3: dark green; DE10: dark purple; CA2: #; VF6: ***. The numbered blocks are mountain passes presenting possible cross‐alpine colonization points: 1 = Simplonpass, 2 = Lukmanierpass, 3 = San Bernardino Pass, 4 = Malojapass, 5 = Bernina Pass, 6 = Reschenpass

The same phylogenetic relationships were recovered using ML and Bayesian approaches, as well as among the *cytb1*, COXI, and concatenated datasets, and were concordant with previously published phylogenies (Figure 2a; Veith et al., 2003; Vences et al., 2013). A basal split in the *R*. *temporaria* clade separates three Spanish clades that occur exclusively in Iberia (Vences et al., 2013) from the rest of the samples. *R*. *temporaria* is further split into an eastern and a western clade with high posterior probability (0.99). Within the western clade, a further split is found between populations from southern Switzerland and all other western populations (including northern Switzerland/Western Europe; posterior probability = 0.97).

The estimated mean coalescent times in Figure 2a support a divergence between the eastern and western clades at ~210 kya, and a more recent divergence between CH‐South and the rest of the western clade at ~110 kya.

### 3.3 Origin of haplotypes in Switzerland

Phylogenetic analysis based on 569 bp of COXI revealed that haplotypes from the Italian Alpine Lineage I (API) described in Stefani et al. (2012) grouped with CH‐South haplotypes with high posterior probability (0.94; Figure 2b). Three haplotypes identified by Stefani et al. (2012)—FN813810.1, FN813785.1, and FN813786—were identical to CH‐South haplotypes. API occurs in the central Italian Alps and contains the most abundant Italian Alpine haplotype (CA2), which was identical to CHS03. In our sample, this haplotype was found only in a population on a high pass in southern Switzerland, on the border with Italy. The most widespread CH‐South haplotype was identical to the widespread API haplotype DE10. A third haplotype (API: VF6) was identical to CHS06, found only at high elevation (>1800 m) in southwestern Switzerland (Figure 2b,c).

The northern Swiss lineage (CH‐North) clustered with APIII with high posterior probability (1.0) based on the Bayesian phylogenetic analysis. One previously identified haplotype from APIII (FN813809.1 = VF3) was identical to the most common CH‐North haplotype (CHN01). Geographically, APIII and VF3 were found in northwestern Italy, but in Switzerland CHN01 occurred mostly north of the Alpine ridge (Figure 2c). APIII clustered with a haplotype from southern France with high posterior probability (0.93; Figure 2b), which suggests that this haplogroup is analogous to the green haplogroup described by Vences et al. (2013) and is widespread across France, Germany, and Great Britain.

### 3.4 Demographic history

The mismatch distribution analyses did not converge for CH‐North for either of the mitochondrial genes or the combined dataset, but Tajima's *D*, Fu and Li's *D*, and Fu's *F* were negative and significant for all CH‐North datasets indicative of a population expansion (Table 1). Tajima's *D*, Fu and Li's *D*, and Fu's *F* were non‐significant for CH‐South, and there was no evidence for population expansion for any of the CH‐South analyses.

## 4 DISCUSSION

These results may indicate that *Rana temporaria* recolonized Western Europe almost exclusively from multiple refugia in the Italian Alps. Populations originating from hypothesized refugia in the Iberian Peninsula occur only in Spain and southwestern France. Our fine‐scale sampling of populations across Switzerland provides evidence of trans‐Alpine colonization from the central Italian Alps into Switzerland, and recolonization from the western Italian Alps around the Alpine arc into much of Western Europe. We found two deeply diverged mitochondrial lineages occurring roughly north and south of the northern Alps, with contact zones in eastern and western Switzerland. These results strongly suggest that the Alps did not present a significant barrier to dispersal for this species during the recolonization of Europe. This has resulted in complex regional variation in the genetic structure of *R*. *temporaria* across Switzerland, which could have important implications for population persistence and adaptive potential. Importantly, our analyses are based on mitochondrial markers, and mitonuclear discordance is known to be prevalent in phylogeographic studies due to gene flow, incomplete lineage sorting, or confounded taxonomies (e.g., Chen et al., 2020; Firneno et al., 2020). Thus, further studies on *R*. *temporaria* should include nuclear DNA markers and more extensive geographic sampling to corroborate the results presented here.

### 4.1 Eastern and Western European lineages

Consistent with previous research, we found a deep phylogenetic split between eastern and Western European lineages of *R*. *temporaria* (Palo et al., 2004). The age of the east–west divergence, calibrated based on the divergence time of *R*. *dalmatina* (3.18 Mya; Veith et al., 2003), was placed in the late Pleistocene (~0.215 Mya, during the Riss Glaciation). This same divergence was previously estimated as ~0.71 Mya (CI 0.53–0.95 Mya) based on *cytb* sequences (Palo et al., 2004), corresponding to the Gunz Glaciation during the first set of glacial cycles in the early Pleistocene (ending roughly 0.4 Mya). The discrepancy may be attributed to the large confidence intervals associated with the statistical priors in our analyses, as well as the slightly different calibration points included in both studies. However, our estimated date and that of Palo et al. (2004) both suggest that the divergence between the eastern and western clades is attributable to dramatic climatic cycles during the Pleistocene, which began ~2.6 Mya (Gibbard et al., 2010). Importantly, several different divergence times have been proposed between brown frogs, and a consensus has not yet been reached. For example, a much older divergence time of 4.1 Mya (compared to 1.12 Mya used in this study; Veith et al., 2003) has recently been estimated between *R*. *parvipalmata* and *R*. *temporaria* based on genome‐wide nuclear phylogenies (Dufresnes et al., 2021). Thus, the timescale of divergence between the eastern and western clades may have occurred much earlier than estimated here.

Two previous studies disagreed about the existence of a contact zone between the eastern and western lineages in northern Switzerland and along the French Mediterranean into Spain (Rodrigues et al., 2017; Teacher et al., 2009). Our thorough sample of northern Switzerland, including several populations in the lowlands where the contact zone was said to lie (Teacher et al., 2009), uncovered no eastern haplotypes whatsoever, and hence no evidence of contact between clades. Presumably, a secondary contact zone between the eastern and western haplogroups occurs farther to the east.

### 4.2 Post‐glacial recolonization of Western Europe

Several studies have identified multiple glacial refugia in the Italian peninsula that consequently led to multiple deeply diverged lineages (Canestrelli et al., 2006, 2008; Canestrelli & Nascetti, 2008; Crottini et al., 2007). Our results instead support the assertion that *R*. *temporaria* recolonized Western Europe from multiple sub‐refugia within the Italian refugium (Stefani et al., 2012). We identified two western mitochondrial lineages distributed roughly north and south of the northern Alps within Switzerland. The two lineages are estimated to have diverged ~110 kya, at the onset of the most recent glacial cold period. Four Alpine lineages have been described in northern Italy, two of which (API and APIII) are distributed south of Switzerland roughly east and west of the Ticino valley (Figure 2c). Our phylogenetic analyses suggest that the southern (CH‐South) haplogroup most likely originates from API in the central Italian Alps, while the northern (CH‐North) haplogroup originates from APIII.

**FIGURE 3:**
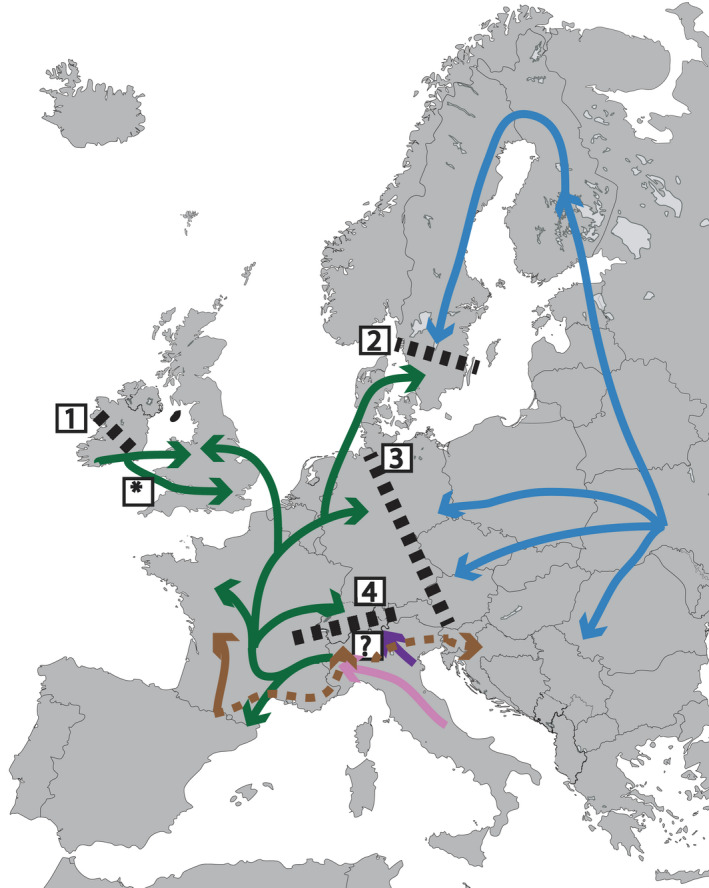
Proposed *R. temporaria* post‐glacial recolonization routes indicated by colored arrows of haplogroups of the same color in Figure 1b. Dashed lines show probable regions of secondary contact between diverged lineages. 1 = common western European lineage meets *R. temporaria* from the Irish refugium (Teacher et al., 2009); 2 = likely original contact zone between eastern and western haplogroup, but only the western haplogroup is found here currently (Palo et al., 2004); 3 = contact zone between eastern and western haplogroups in northern Germany (Schmeller et al., 2008), but contact zone south of this site has not been described; 4 = contact zone between CH‐South and CH‐North described in this paper; ? = possible contact zone between the Iberian (CH‐brown) and Italian lineages described in this paper. The asterisk (*) denotes an alternative origin and colonization route of the green haplotype from the Irish refugium into western Europe

Geographic analysis of the CH‐North and CH‐South lineages suggests that they recolonized Western Europe via two different routes (Figure 3). The geographic distribution of the southern lineage across the central Alpine ridge is evidence of multiple trans‐Alpine colonizations into Switzerland (Figure 2c). Three CH‐South haplotypes were identical to API haplotypes, and their geographic distribution can be used to narrow down colonization routes. Assuming a conservative maximum habitable elevation of about 2000 m (as in Braaker & Heckel, 2009), there are six possible passes across the central Alpine ridge (Figure 2c). The most widespread API haplotype (CA2) was identical to a CH‐South haplotype found in southeastern Switzerland, which suggests colonization across the Maloja Pass (4, Figure 2c) or Bernina Pass (5, Figure 2c).

Southwestern Switzerland contained several sites with secondary contact between API and APIII, which could suggest a southern trans‐Alpine colonization route of APIII into Switzerland. Simplonpass (1, Figure 2c) presents a possible route in the west, but the most likely point of colonization is difficult to identify because the most common CH‐South haplotype (API haplotype DE10) was geographically widespread in northern Italy.

In other European taxa, studies of trans‐Alpine colonization have identified the same routes for both plants and animals (Braaker & Heckel, 2009; Lugon‐Moulin & Hausser, 2002; Parisod, 2008; Yannic et al., 2008, 2012). The example of European white oaks (Mátyás & Sperisen, 2001), which presently occur only rarely above 1200 m in the Alps (Rigling et al., 2013), illustrates that trans‐Alpine recolonization was not limited only to taxa adapted to cold climates. Presumably, dispersal over Alpine passes was more extensive when the treeline was ~200 m higher than it is today, which occurred regularly during the period of 5000–10,000 years ago (e.g., Ammann et al., 2000; Heiri et al., 2006; Tinner & Theurillat, 2003). It may be possible to infer exact colonization routes using nuclear markers and fine‐scale geographic sampling of populations across the passes potentially used for colonization.

CH‐North haplotypes appear to have colonized Switzerland up the Rhone River from the west or from the northwest over the Jura Mountains (Figure 3). The geographic distribution of the derived CH‐North haplotypes toward southern Switzerland (light green haplotypes; Figure 1a) and overall low genetic diversity (Table 1) suggest that this lineage expanded southwards in Switzerland rather than colonizing via trans‐Alpine routes as with the CH‐South lineage. Three possible origins of this haplogroup have been proposed: Iberia, a cryptic refugium in southern Ireland, and northern Italy (Stefani et al., 2012; Teacher et al., 2009; Vences et al., 2013). Although we cannot definitively rule out Iberia or Ireland, our results suggest an Italian origin. Specifically, the common Western European haplogroup (T4; Vences et al., 2013) occupies northern Switzerland, and we found that this haplogroup is identical to Italian APIII found in the northwestern Italian Alps. In addition, the rest of the CH‐North haplotypes form a monophyletic group with APIII (Figure 2b). Finally, genetic diversity is expected to decrease with distance from glacial refugia as populations experience bottleneck events with colonization (Hewitt, 1996). The genetic variation in the green/APII/T4 haplogroup is substantially higher in northern Italy than in Ireland or Iberia, suggesting that this was the main refugium of the haplogroup.

The identification of a second CH‐brown lineage exclusively south of the central Alpine ridge in southern Switzerland suggests that there may be eastward expansion from Iberia south of the Alps (Figures 1 and 3). This lineage is ubiquitous in the Pyrenees, and is found in southern France, northern Italy, and Croatia (Vences et al., 2013). Deglaciation of the Tinee Valley on the border between France and Italy occurred during the Oldest Dryas (~15 kya), earlier than in the rest of the Alps where glaciers retreated during the Younger Dryas (~11 kya) and the late ice retreat (~9 kya) (Darnault et al., 2012). This potential early colonization corridor could have provided the way for an eastward expansion from the Pyrenees, across northern Italy, to Croatia. Although westward expansion from the Carpathian refugium through northern Italy has been described (Demesure et al., 1996; Taberlet et al., 1998), we found no examples of an eastward expansion from Iberia into northern Italy. Longer mtDNA fragments and more extensive sampling would be needed to investigate this unusual colonization route further.

### 4.3 Alpine topography affects genetic diversity in Switzerland

While the Alps presented an important barrier to dispersal into Europe for many species, they led to complex colonization histories for a high‐elevation and cold‐tolerant species. This has resulted in unusually high levels of genetic diversity where lineages from multiple refugia meet in Switzerland. For example, several lineages of *Arabis halleri* are found across the Alps, which have resulted in different loci associated with adaptation to environmental conditions in neighboring valleys (Rellstab et al., 2016). A similarly high degree of genetic diversity is found in Oak species (Mátyás & Sperisen, 2001). Although fewer, there are also examples of animals that have colonized Switzerland across the Alps. For example, there is evidence that grass snakes (*Natrix* sp.) have colonized southern Switzerland via multiple routes from multiple Italian refugia (Kindler & Fritz, 2018), as has the Alpine vole (Braaker & Heckel, 2009) and the common shrew (Yannic et al., 2008).

For *R*. *temporaria*, this pattern of increased diversity extends to *Dmrt1* sex determining haplotypes (Phillips et al., 2020). Sex determination in *R*. *temporaria* is linked to differentiation on the homomorphic sex chromosome (chromosome pair 1; Chr01), with several Y‐specific alleles located along the chromosome. Males can be completely determined by differentiation at these loci (XY males), or they can be completely undifferentiated at the sex chromosome (XX males). Intermediate males (XY^o^ males) are only differentiated at the *Dmrt1* locus (Ma et al., 2016; Rodrigues et al., 2017), a highly conserved transcription factor associated with testes formation and masculinization in various vertebrate species, including several frog species (Brelsford et al., 2016). Two *Dmrt1* haplotypes have been found in Switzerland (Phillips et al., 2020): haplotype Y_A_ produces well‐differentiated sex chromosomes and is found south of the Alpine ridge, matching mitochondrial haplotype CH‐South, while Y_B_ is associated with relatively undifferentiated sex chromosomes and coincides with the distribution of mitochondrial haplotype CH‐North. The study provides evidence that the geographic distribution of sex haplotypes is determined by phylogeographic range expansion, rather than by climate, providing a powerful example of how colonization history in the Alps can determine and maintain diversity.

## 5 CONCLUSION

The *R*. *temporaria* western mitochondrial clade is exceptional in that it harbors more genetic diversity than any other European brown frog (Palo et al., 2004; Vences et al., 2013). We show that much of this variation can be attributed to the European Alps. The topographic complexity of the Alps has resulted in multiple glacial refugia and subsequent divergence between *R*. *temporaria* lineages. After the retreat of the glaciers, the Alps presented a semi‐permeable barrier to dispersal for these lineages, resulting in multiple colonization events from the south. There was simultaneous colonization from other refugia in northern Italy, following a circum‐Alpine route to colonize Western Europe and Switzerland (Figure 3). This has resulted in two deeply diverged lineages that occur in the north and south of Switzerland with contact zones in the east and west. Our work highlights how fine‐scale phylogeographic studies across the Alps can elucidate the phylogeographic history of cold‐adapted European taxa.

## CONFLICT OF INTEREST

None declared.

## AUTHOR CONTRIBUTIONS

**Alexandra Jansen van Rensburg:** Conceptualization (lead); Data curation (lead); Formal analysis (lead); Funding acquisition (equal); Investigation (lead); Methodology (lead); Writing‐original draft (lead). **Mathieu Robin:** Data curation (supporting); Formal analysis (supporting); Writing‐review & editing (supporting). **Barret Phillips:** Data curation (supporting); Formal analysis (supporting); Writing‐review & editing (supporting). **Josh Van Buskirk:** Data curation (supporting); Funding acquisition (lead); Project administration (lead); Writing‐review & editing (supporting).

## Supporting information

Figure S1

Figure S2

Figure S3

Tables S1‐S3

## Data Availability

All sequences are available on NCBI. Accession numbers for COXI: MF624310–MF624324; *cytb*: MF624327–MF624355. Further data are available on Dryad: https://doi.org/10.5061/dryad.2z34tmpnh.
