# Supplementary material for: European common frog (Rana temporaria) recolonized Switzerland from multiple glacial refugia in northern Italy via trans‐ and circum‐Alpine routes
Source: Ecol Evol. 2021 Nov 2;11(22):15984–94. doi: 10.1002/ece3.8268 (PMC8601898; doi:10.1002/ece3.8268)
Supplement: Supplementary file 4 — Tables S1‐S3 [file ECE3-11-15984-s001.docx]

***Appendix***

***Table S1*** Populations sampled and sequenced at cytochrome b. N= number of samples sequenced, Haplotypes= number of haplotypes found in the population, Hd (SD)= Haplotype diversity (standard deviation), nd (SD) = nucleotide diversity (standard deviation).

| **Population** | **Lat.** | **Long.** | **Elevation** | **N** | **length (bp)** | **Haplotypes** | **Hd (SD)** | **nd (SD)** |
| --- | --- | --- | --- | --- | --- | --- | --- | --- |
| agra | 46.03 | 8.90 | 930 | 6 | 448 | 3 | 0.8 (0.122) | 0.00238 (0.0005) |
| alpl | 46.94 | 8.66 | 1506 | 4 | 448 | 2 | 0.5 (0.27) | 0.00112 (0.00059) |
| apla | 46.79 | 9.50 | 2118 | 6 | 448 | 3 | 0.6 (0.22) | 0.0091 (0.00297) |
| arce | 46.16 | 8.75 | 408 | 6 | 448 | 3 | 0.73 (0.16) | 0.0064 (0.00251) |
| bach | 46.67 | 8.03 | 2265 | 6 | 448 | 2 | 0.53 (0.17) | 0.00119 (0.00038) |
| bela | 46.38 | 7.98 | 2179 | 6 | 448 | 3 | 0.73 (0.16) | 0.00699 (0.00288) |
| bend | 46.49 | 6.88 | 590 | 6 | 448 | 1 | 0 (0) | 0 (0) |
| berm | 47.02 | 7.52 | 550 | 6 | 448 | 1 | 0 (0) | 0 (0) |
| bide | 47.14 | 9.40 | 1983 | 6 | 448 | 1 | 0 (0) | 0 (0) |
| birk | 47.30 | 8.81 | 550 | 4 | 448 | 1 | 0 (0) | 0 (0) |
| bnnp | 46.41 | 10.03 | 2342 | 6 | 448 | 3 | 0.73 (0.16) | 0.00193 (0.00055) |
| buel | 46.86 | 9.73 | 2260 | 6 | 448 | 2 | 0.33 (0.22) | 0.00074 (0.00048) |
| cava | 46.36 | 9.03 | 2003 | 6 | 448 | 2 | 0.33 (0.22) | 0.00521 (0.00336) |
| csan | 46.32 | 7.30 | 2110 | 6 | 448 | 1 | 0 (0) | 0 (0) |
| egel | 47.25 | 9.50 | 445 | 6 | 448 | 2 | 0.33 (0.22) | 0.00595 (0.00384) |
| fada | 46.98 | 9.60 | 1093 | 3 | 448 | 2 | 0.67 (0.31) | 0.00149 (0.00070) |
| fess | 47.02 | 9.14 | 2184 | 4 | 448 | 2 | 0.5 (0.27) | 0.0012 (0.00059) |
| flue | 46.75 | 9.95 | 2388 | 3 | 448 | 3 | 0.83 (0.05) | 0.0115 (0.00335) |
| forn | 46.48 | 8.58 | 2089 | 5 | 448 | 3 | 0.7 (0.22) | 0.00982 (0.00295) |
| full | 46.17 | 7.10 | 2074 | 5 | 448 | 3 | 0.7 (0.22) | 0.00759 (0.00333) |
| fuor | 46.44 | 9.83 | 2494 | 6 | 448 | 1 | 0 (0) | 0 (0) |
| gdwe | 46.64 | 8.07 | 1380 | 6 | 448 | 1 | 0 (0) | 0 (0) |
| gola | 46.10 | 8.97 | 975 | 6 | 448 | 5 | 0.93 (0.12) | 0.00774 (0.00282) |
| gott | 46.59 | 8.56 | 2116 | 6 | 448 | 2 | 0.33 (0.22) | 0.00149 (0.00096) |
| grma | 46.24 | 7.01 | 425 | 6 | 448 | 3 | 0.6 (0.22) | 0.00714 (0.00356) |
| grsh | 46.66 | 8.10 | 1929 | 6 | 448 | 3 | 0.73 (0.16) | 0.00193 (0.00055) |
| gruu | 46.86 | 9.79 | 2120 | 4 | 448 | 1 | 0 (0) | 0 (0) |
| hdns | 46.85 | 9.76 | 1918 | 4 | 448 | 1 | 0 (0) | 0 (0) |
| jagg | 46.74 | 8.06 | 570 | 4 | 448 | 1 | 0 (0) | 0 (0) |
| kand | 46.63 | 7.69 | 698 | 4 | 448 | 1 | 0 (0) | 0 (0) |
| kebe | 47.54 | 8.78 | 453 | 4 | 448 | 2 | 0.5 (0.27) | 0.00112 (0.00059) |
| lens | 46.29 | 7.46 | 1338 | 5 | 448 | 2 | 0.4 (0.24) | 0.00179 (0.00106) |
| lucm | 46.56 | 8.80 | 1922 | 6 | 448 | 1 | 0 (0) | 0 (0) |
| magn | 46.43 | 8.68 | 1840 | 4 | 448 | 2 | 0.5 (0.27) | 0.0067 (0.00355) |
| mart | 46.03 | 8.94 | 407 | 5 | 448 | 2 | 0.6 (0.18) | 0.00134 (0.00039) |
| mctn | 46.48 | 9.72 | 2542 | 5 | 448 | 1 | 0 (0) | 0 (0) |
| mgns | 46.25 | 6.85 | 1372 | 4 | 448 | 2 | 0.5 (0.27) | 0.00112 (0.00059) |
| moau | 46.34 | 6.79 | 1850 | 6 | 448 | 2 | 0.53 (0.17) | 0.00119 (0.00038) |
| moir | 46.10 | 7.57 | 2553 | 4 | 448 | 1 | 0 (0) | 0 (0) |
| muet | 47.45 | 8.61 | 460 | 6 | 448 | 2 | 0.53 (0.17) | 0.00119 (0.00038) |
| munt | 46.73 | 9.44 | 648 | 2 | 448 | 2 | 1 (0.5) | 0.00223 (0.00112) |
| oalp | 46.65 | 8.64 | 1960 | 6 | 448 | 3 | 0.73 (0.16) | 0.00208 (0.00058) |
| otte | 46.73 | 7.36 | 1455 | 6 | 448 | 1 | 0 (0) | 0 (0) |
| petl | 45.89 | 7.15 | 2655 | 4 | 448 | 1 | 0 (0) | 0 (0) |
| pizo | 46.98 | 9.42 | 2209 | 4 | 448 | 1 | 0 (0) | 0 (0) |
| pozz | 46.35 | 8.96 | 290 | 4 | 448 | 3 | 0.83 (0.22) | 0.00223 (0.00076) |
| prad | 46.78 | 9.53 | 1449 | 5 | 448 | 4 | 0.9 (0.16) | 0.01161 (0.00307) |
| roes | 46.90 | 7.20 | 538 | 6 | 448 | 1 | 0 (0) | 0 (0) |
| rose | 46.14 | 7.05 | 450 | 4 | 448 | 3 | 0.83 (0.22) | 0.01042 (0.00367) |
| rotc | 47.01 | 9.31 | 2185 | 4 | 448 | 1 | 0 (0) | 0 (0) |
| rusc | 46.37 | 7.24 | 1315 | 4 | 448 | 1 | 0 (0) | 0 (0) |
| sali | 46.26 | 8.69 | 331 | 4 | 448 | 3 | 0.83 (0.22) | 0.00967 (0.00383) |
| saxm | 47.07 | 9.38 | 463 | 9 | 448 | 1 | 0 (0) | 0 (0) |
| scai | 45.98 | 8.93 | 298 | 2 | 448 | 1 | 0 (0) | 0 (0) |
| seeo | 47.05 | 9.58 | 2030 | 6 | 448 | 1 | 0 (0) | 0 (0) |
| seji | 46.80 | 9.73 | 2090 | 6 | 448 | 2 | 0.83 (0.22) | 0.00595 (0.00384) |
| shwe | 47.19 | 9.33 | 1159 | 6 | 448 | 1 | 0 (0) | 0 (0) |
| siec | 46.99 | 9.55 | 517 | 5 | 448 | 2 | 0.6 (0.18) | 0.00938 (0.00274) |
| star | 46.27 | 8.77 | 1892 | 6 | 448 | 2 | 0.53 (0.17) | 0.00119 (0.00038) |
| stba | 46.49 | 9.17 | 2059 | 6 | 448 | 2 | 0.33 (0.22) | 0.00521 (0.00336) |
| stir | 46.53 | 7.55 | 2070 | 4 | 448 | 1 | 0 (0) | 0 (0) |
| stls | 46.97 | 9.75 | 1672 | 6 | 448 | 2 | 0.33 (0.22) | 0.00521 (0.00336) |
| tana | 46.35 | 6.84 | 1408 | 6 | 448 | 3 | 0.6 (0.22) | 0.00149 (0.00062) |
| trim | 46.90 | 9.54 | 545 | 5 | 448 | 2 | 0.4 (0.24) | 0.00625 (0.00371) |
| trpa | 46.29 | 7.28 | 2196 | 4 | 448 | 1 | 0 (0) | 0 (0) |
| tsee | 46.55 | 7.75 | 1151 | 6 | 448 | 1 | 0 (0) | 0 (0) |
| vilt | 47.03 | 9.45 | 486 | 4 | 448 | 2 | 0.5 (0.27) | 0.00112 (0.00059) |
| vora | 47.16 | 9.38 | 1123 | 5 | 448 | 2 | 0.4 (0.24) | 0.00089 (0.00053) |
| wale | 47.12 | 9.10 | 427 | 6 | 448 | 2 | 0.33 (0.22) | 0.00074 (0.00048) |
| wdji | 46.81 | 9.72 | 1638 | 6 | 448 | 3 | 0.8 (0.12) | 0.01071 (0.00303) |
| wise | 47.19 | 9.48 | 445 | 5 | 448 | 4 | 0.9 (0.16) | 0.01027 (0.00247) |
| zeni | 46.52 | 8.89 | 1397 | 5 | 448 | 1 | 0 (0) | 0 (0) |

***Table S2*** Additional populations for which 331bp cytochrome b sequences were generated to incorporate with the dataset from Switzerland. Name=corresponds to names in Figure S3; Stefani *et al.* 2012 pops = tissue samples from the populations described in their study shared with us by the authors to sequence the portion of the cytochrome b gene used in the rest of our study; N=Number of samples sequenced; Haplotypes=number of haplotypes found in the population.

| **Name** | **Population** | **Stefani *et al.* 2012 pops** | **Country** | **Lat** | **Long** | **N** | **length (bp)** | **Haplotypes** |
| --- | --- | --- | --- | --- | --- | --- | --- | --- |
| djer | Djerdap | - | Serbia | 44.39 | 22.18 | 12 | 331 | 1 |
| loca | Locana | - | Italy | 45.43 | 7.43 | 3 | 331 | 1 |
| grei | Greifswald | - | Germany | 54.08 | 13.44 | 7 | 331 | 1 |
| deve | A. Devero | Pop 8 | Italy | 46.32 | 8.26 | 3 | 331 | 1 |
| pora | M. Pora | Pop 9 | Italy | 45.88 | 10.10 | 3 | 331 | 3 |
| scat | S. Caterina | Pop 11 | Italy | 46.41 | 10.49 | 3 | 331 | 2 |
| vfer | V. Ferret | Pop 13 | Italy | 45.85 | 7.02 | 3 | 331 | 2 |
| trav | Traversella | Pop 14 | Italy | 45.46 | 7.32 | 3 | 331 | 1 |
| issi | Issiglio | Pop 15 | Italy | 45.46 | 7.32 | 3 | 331 | 1 |

***Table S3*** Populations sampled and sequenced at COXI. N = number of samples sequenced, Haplotypes = number of haplotypes found in the population, Hd (SD) = Haplotype diversity (standard deviation), nd (SD) = nucleotide diversity (standard deviation).

| **Population** | **N** | **length (bp)** | **nr haplotypes** | **Hd (SD)** | **nucleotide diversity (SD)** |
| --- | --- | --- | --- | --- | --- |
| alpl | 4 | 628 | 1 | 0 (0) | 0 (0) |
| arce | 3 | 628 | 1 | 0 (0) | 0 (0) |
| bach | 4 | 628 | 1 | 0 (0) | 0 (0) |
| bela | 3 | 628 | 1 | 0 (0) | 0 (0) |
| bide | 3 | 628 | 2 | 0.67 (0.31) | 0.00106 (0.00050) |
| birk | 4 | 628 | 1 | 0 (0) | 0 (0) |
| bnnp | 3 | 628 | 2 | 0.67 (0.31) | 0.00106 (0.00050) |
| cava | 3 | 628 | 2 | 0.67 (0.31) | 0.00637 (0.003) |
| egel | 3 | 628 | 2 | 0.67 (0.31) | 0.00106 (0.00050) |
| fada | 3 | 628 | 1 | 0 (0) | 0 (0) |
| flue | 4 | 628 | 3 | 0.83 (0.22) | 0.00717 (0.00207) |
| forn | 4 | 628 | 3 | 0.83 (0.22) | 0.00717 (0.00207) |
| fuor | 3 | 628 | 1 | 0 (0) | 0 (0) |
| gott | 4 | 628 | 2 | 0.5 (0.27) | 0.0008 (0.00042) |
| grma | 4 | 628 | 2 | 0.5 (0.27) | 0.0008 (0.00042) |
| grsh | 4 | 628 | 1 | 0 (0) | 0 (0) |
| gruu | 4 | 628 | 2 | 0.5 (0.27) | 0.0008 (0.00042) |
| hdns | 4 | 628 | 2 | 0.5 (0.27) | 0.0008 (0.00042) |
| jagg | 4 | 628 | 1 | 0 (0) | 0 (0) |
| kand | 4 | 628 | 2 | 0.5 (0.27) | 0.00159 (0.00084) |
| kebe | 4 | 628 | 1 | 0 (0) | 0 (0) |
| magn | 3 | 628 | 2 | 0.67 (0.31) | 0.00743 (0.0035) |
| mart | 4 | 628 | 2 | 0.67 (0.2) | 0.00212 (0.00065) |
| mctn | 4 | 628 | 1 | 0 (0) | 0 (0) |
| mgns | 4 | 628 | 1 | 0 (0) | 0 (0) |
| moir | 4 | 628 | 1 | 0 (0) | 0 (0) |
| petl | 4 | 628 | 1 | 0 (0) | 0 (0) |
| pizo | 4 | 628 | 1 | 0 (0) | 0 (0) |
| pozz | 4 | 628 | 2 | 0.5 (0.27) | 0.00159 (0.00084) |
| prad | 4 | 628 | 2 | 0.5 (0.27) | 0.00478 (0.00253) |
| rotc | 4 | 628 | 1 | 0 (0) | 0 (0) |
| rusc | 4 | 628 | 1 | 0 (0) | 0 (0) |
| seeo | 4 | 628 | 2 | 0.67 (0.2) | 0.00106 (0.00033) |
| shwe | 4 | 628 | 1 | 0 (0) | 0 (0) |
| siec | 1 | 628 | 1 | 0 (0) | 0 (0) |
| star | 1 | 628 | 1 | 0 (0) | 0 (0) |
| stba | 3 | 628 | 3 | 1.0 (0.27) | 0.00743 (0.00304) |
| stir | 2 | 628 | 1 | 0 (0) | 0 (0) |
| stls | 1 | 628 | 1 | 0 (0) | 0 (0) |
| tana | 3 | 628 | 1 | 0 (0) | 0 (0) |
| trpa | 4 | 628 | 1 | 0 (0) | 0 (0) |
| vora | 2 | 628 | 1 | 0 (0) | 0 (0) |
| wdji | 4 | 628 | 2 | 0.5 (0.27) | 0.00478 (0.00253) |
| zeni | 3 | 628 | 1 | 0 (0) | 0 (0) |
